# Supplementary material for: Transposable elements and gene expression during the evolution of amniotes
Source: Mob DNA. 2018 Jun 12;9:17. doi: 10.1186/s13100-018-0124-5 (PMC5998507; doi:10.1186/s13100-018-0124-5)
Supplement: Supplementary file 1 — Supplementary Information Additional file contains supplementary figures and tables as referred to in the main body of the paper. (PDF 848 kb) [file 13100_2018_124_MOESM1_ESM.pdf]

Supplementary Information

Transposable elements and gene expression during the evolution of amniotes

Lu Zeng[1], Stephen M. Pederson[2], R. Daniel Kortschak[1], David L. Adelson[1*]

1 School of Biological Sciences, The University of Adelaide, SA 5005, Australia
2 Bioinformatics Hub, The University of Adelaide, SA 5005, Australia

* david.adelson@adelaide.edu.au

List of Figures

S1 **Change in the levels of ortholog gene expression as a function of TE insertion.** 4

S2 **Change in the levels of ortholog gene expression as a function of species-specific TE insertion.** 5

S3 **Change in the levels of ortholog gene expression as a function of non-species specific TE insertion.** 6

S4 **Change in the level of non-ortholog gene expression as a function of TE insertion.** 7

S5 **Change in the level of non-ortholog gene expression as a function of species-specific TE insertion.** 8

S6 **Change in the level of non-ortholog gene expression as a function of non-species specific TE insertion.** 9

S7 **Factorial map of the principal-component analysis of messenger RNA expression levels.** 10

List of Tables

1 **Gene expression dataset.** . . . 11
2 **Assembly dataset.** . . . 15
3 **Comparison of ortholog with ssTE *vs* orthologs with nsTE and ∅ TE.** . . . 15
4 **Comparison of orthologs with nsTE *vs* orthologs with ssTE and ∅ TE.** . . . 15
5 **Comparison of non-orthologs with ssTE *vs* non-orthologs with nsTE and ∅ TE.** . . . 16
6 **Comparison of non-orthologs with nsTE *vs* non-orthologs with ssTE and ∅ TE.** . . . 16
7 **Summary of orthologs/non-orthologs with nsTE, orthologs/non-orthologs with ssTE and orthologs/non-orthologs with ∅ TE.** . . . 16
8 **Difference in the gene expression of orthologs/non-orthologs with a TE insertion.** . . . 17

Supplementary Figures

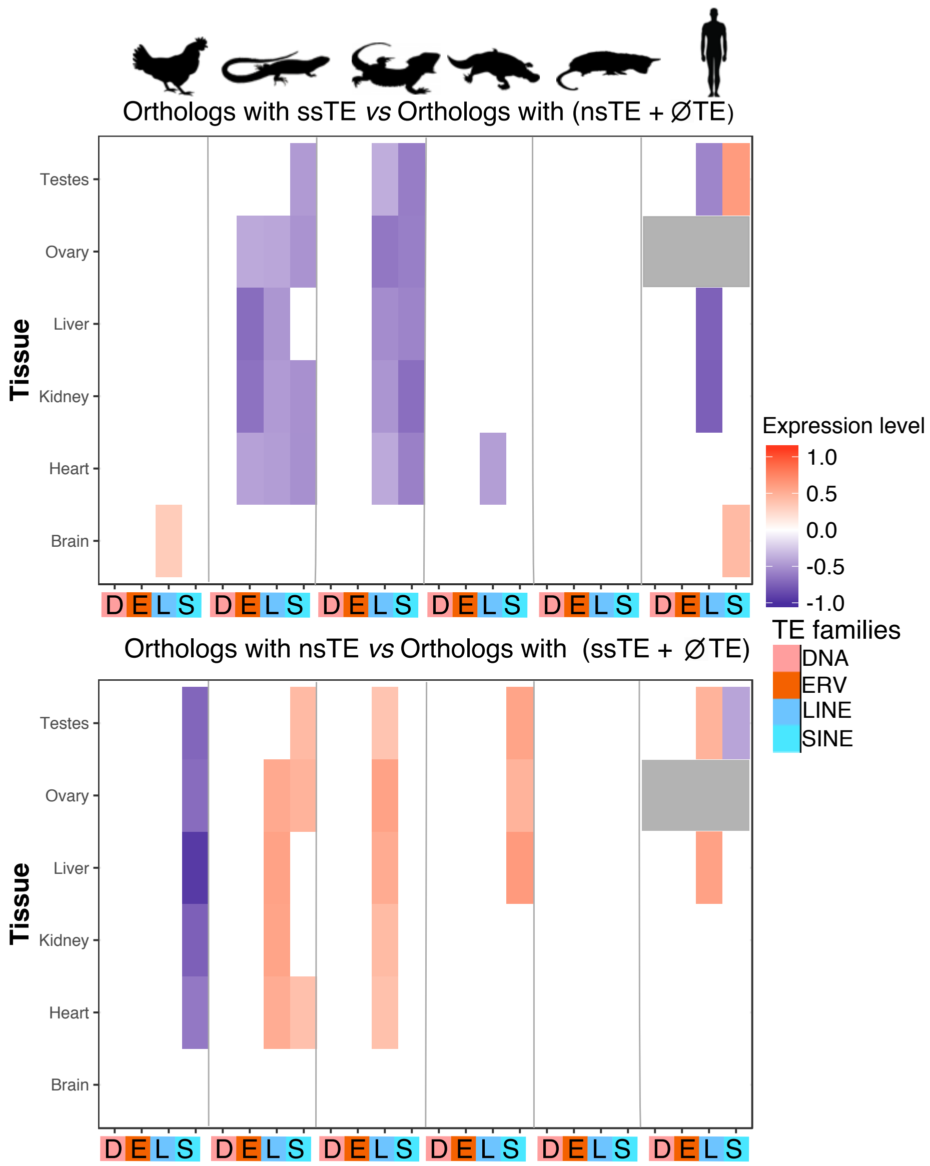

Figure S1: **Change in the levels of ortholog gene expression as a function of TE insertion.** This figure shows the association between ortholog gene expression levels in six species (from left to right: chicken, anole lizard, bearded dragon (pogona), platypus, opossum and human) with recent species-specific TE insertions (ssTE) or non-species specific TE insertions (nsTE) (from left to right: DNA transposons, ERV/LTR, LINE or SINE). A weighted bootstrap approach was used to compare the median gene expression levels of orthologs with a ssTE/nsTE insertion compared to orthologs without ssTE/nsTE. Gene expression levels are log2-transformed. Comparisons without statistically significant gene expression changes are shown in white. Statistically significant increased gene expression shown in red and statistically significant decreased gene expression in blue. Grey shading indicates no samples were available for this comparison.

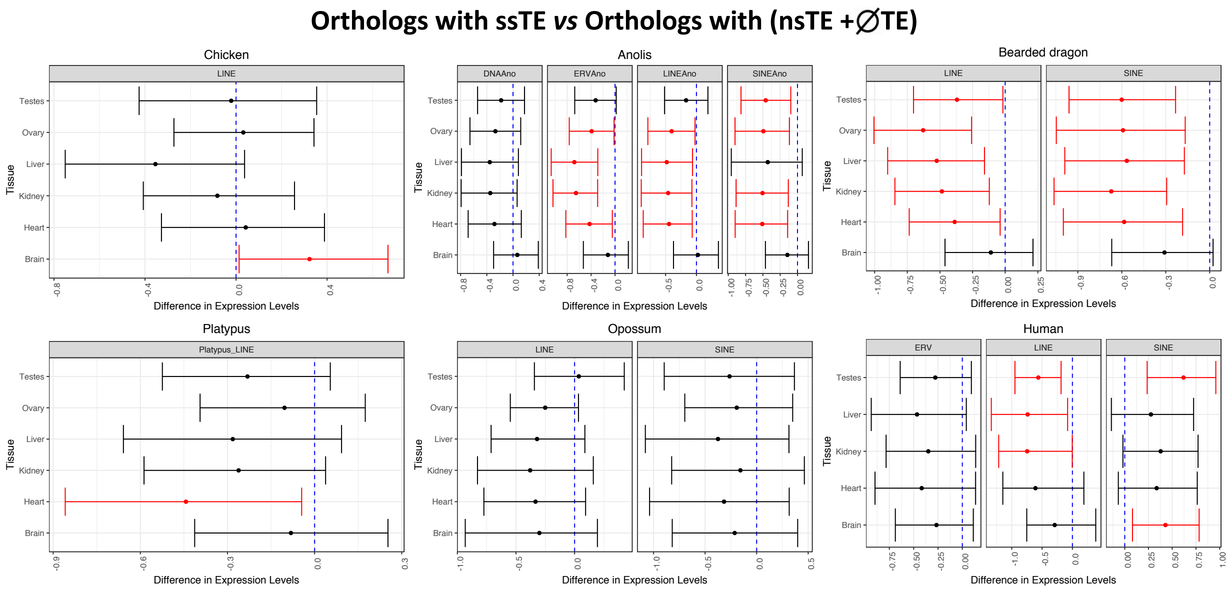

Figure S2: **Change in the levels of ortholog gene expression as a function of species-specific TE insertion.**

This figure shows the association between ortholog median gene expression levels in six species (from left to right: chicken, anole lizard, bearded dragon (pogona), platypus, opossum and human) with recent species-specific TE insertions (ssTE) (from left to right: DNA transposons, ERV/LTR, LINE or SINE). Confidence Intervals for the difference in median log2(TPM) counts. Confidence Intervals were obtained using the weighted bootstrap and are 1-α/m intervals, where α=0.05 and m=nTissues*nElements as the total number of intervals presented. Red dots represent the median value from the bootstrap procedure, whilst the vertical line indicates zero. Intervals which do not contain zero are coloured red, and indicate a rejection of the null hypothesis, H_0: $\Delta\theta$=0, where θ represents the parameter of interest.

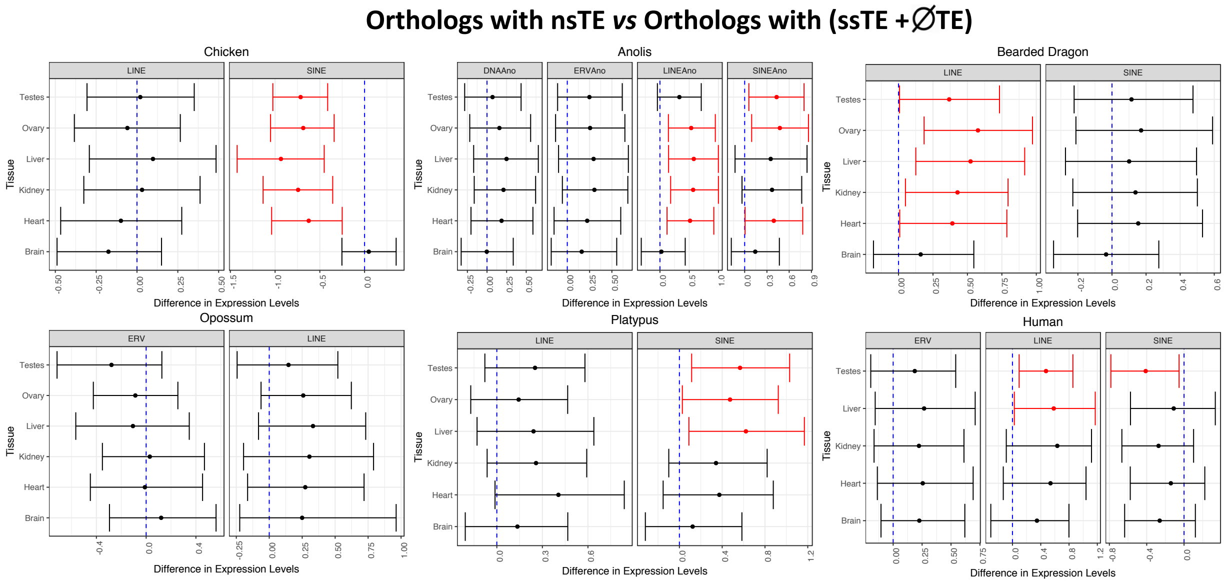

Figure S3: **Change in the levels of ortholog gene expression as a function of non-species specific TE insertion.**
This figure shows the association between ortholog gene expression levels in six species (from left to right: chicken, anole lizard, bearded dragon (pogona), platypus, opossum and human) with non-species specific TE insertions (nsTE) (from left to right: DNA transposons, ERV/LTR, LINE or SINE). Confidence Intervals for the difference in median log2(TPM) counts. Confidence Intervals were obtained using the weighted bootstrap and are 1-α/m intervals, where α=0.05 and m=nTissues*nElements as the total number of intervals presented. Red dots represent the median value from the bootstrap procedure, whilst the vertical line indicates zero. Intervals which do not contain zero are coloured red, and indicate a rejection of the null hypothesis, H_0: $\Delta\theta=0$, where θ represents the parameter of interest.

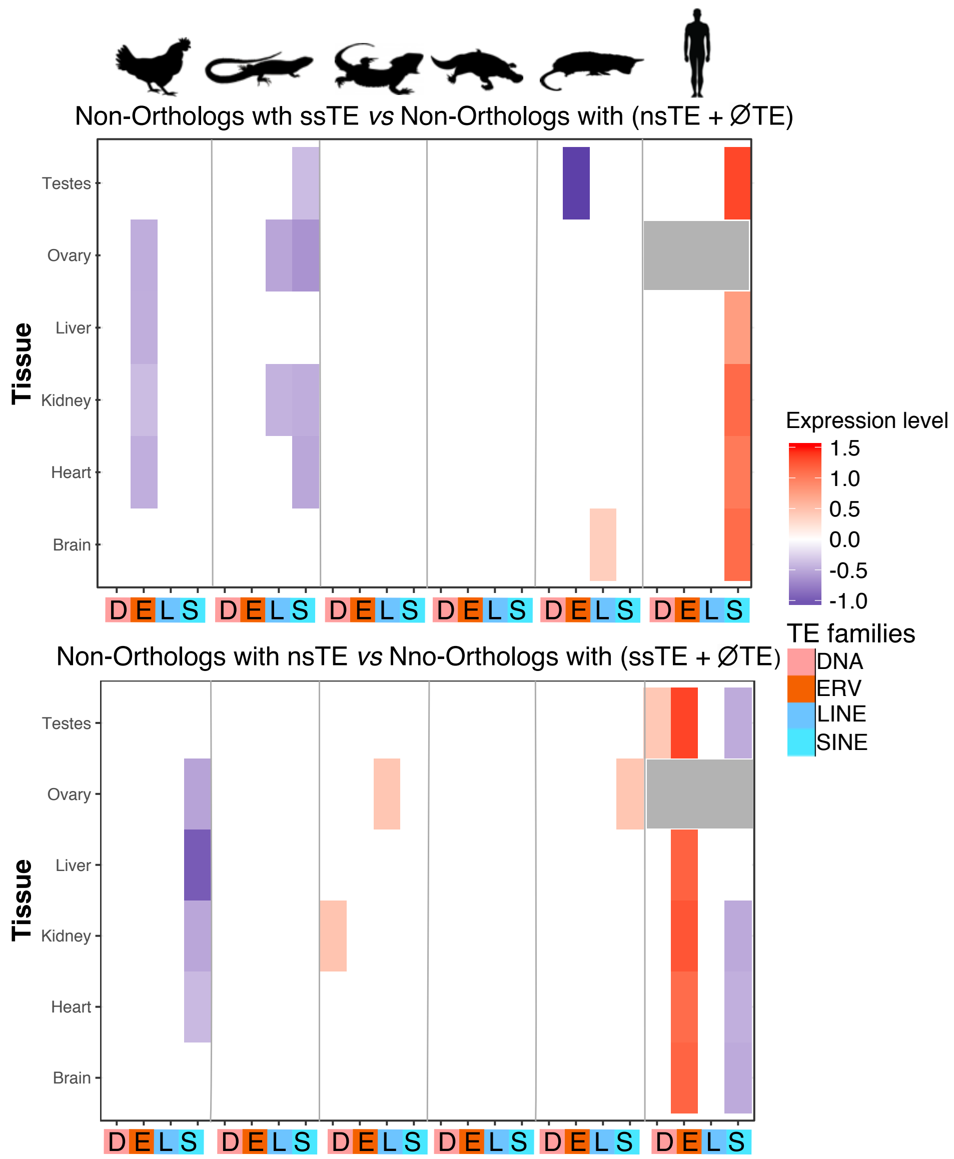

Figure S4: **Change in the level of non-ortholog gene expression as a function of TE insertion.** This figure shows the association between non-ortholog gene expression levels in six species (from left to right: chicken, anole lizard, bearded dragon (pogona), platypus, opossum and human) with recent species-specific TE insertions (ssTE) or non-species specific TE insertions (nsTE) (from left to right: DNA transposons, ERV/LTR, LINE or SINE). A weighted bootstrap approach was used to compare the median gene expression levels of non-orthologous genes with a ssTE/nsTE insertion compared to non-orthologous gene without ssTE/nsTE. Gene expression levels are log2-transformed. Comparisons without statistically significant gene expression changes are shown in white. Statistically significant increased gene expression shown in red and statistically significant decreased gene expression in blue. Grey shading indicates no samples were available for this comparison.

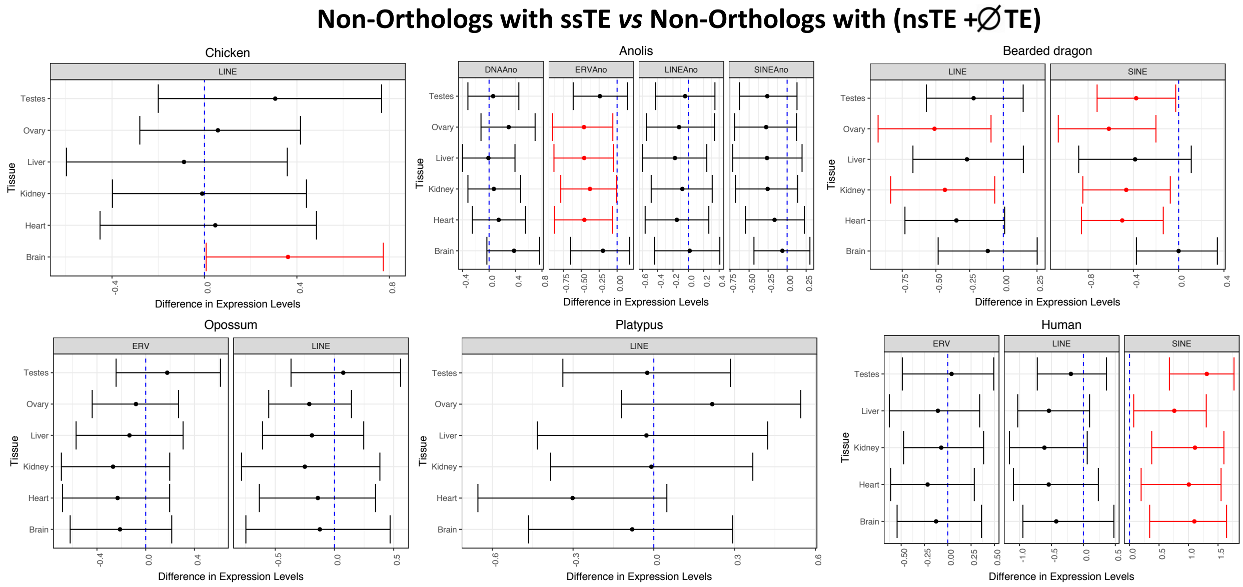

Figure S5: **Change in the level of non-ortholog gene expression as a function of species-specific TE insertion.**

This figure shows the association between non-ortholog gene expression levels in six species (from left to right: chicken, anole lizard, bearded dragon (pogona), platypus, opossum and human) with recent species-specific TE insertions (ssTE) (from left to right: DNA transposons, ERV/LTR, LINE or SINE). Confidence Intervals for the difference in median log2(TPM) counts. Confidence Intervals were obtained using the weighted bootstrap and are 1-α/m intervals, where α=0.05 and m=nTissues*nElements as the total number of intervals presented. Red dots represent the median value from the bootstrap procedure, whilst the vertical line indicates zero. Intervals which do not contain zero are coloured red, and indicate a rejection of the null hypothesis, H_0: $\Delta\theta$=0, where θ represents the parameter of interest.

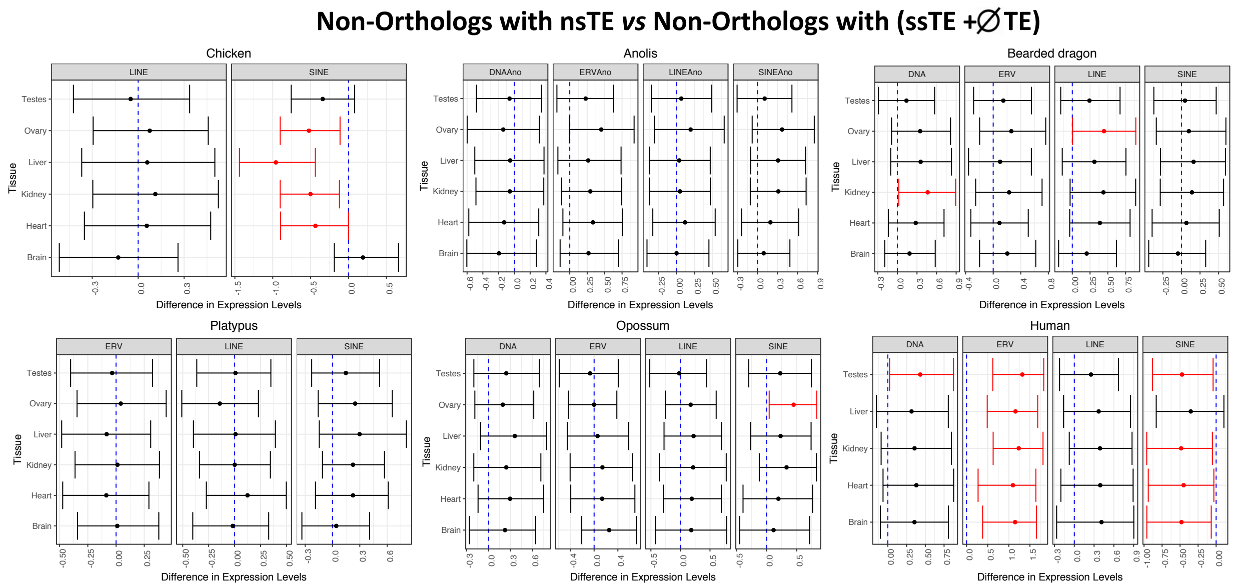

Figure S6: **Change in the level of non-ortholog gene expression as a function of non-species specific TE insertion.**
This figure shows the association between non-ortholog gene expression levels in six species (from left to right: chicken, anole lizard, bearded dragon (pogona), platypus, opossum and human) with non-species specific TE insertions (nsTE) (from left to right: DNA transposons, ERV/LTR, LINE or SINE). Confidence Intervals for the difference in median log2(TPM) counts. Confidence Intervals were obtained using the weighted bootstrap and are 1-α/m intervals, where α=0.05 and m=nTissues*nElements as the total number of intervals presented. Red dots represent the median value from the bootstrap procedure, whilst the vertical line indicates zero. Intervals which do not contain zero are coloured red, and indicate a rejection of the null hypothesis, H_0: $\Delta\theta$=0, where θ represents the parameter of interest.

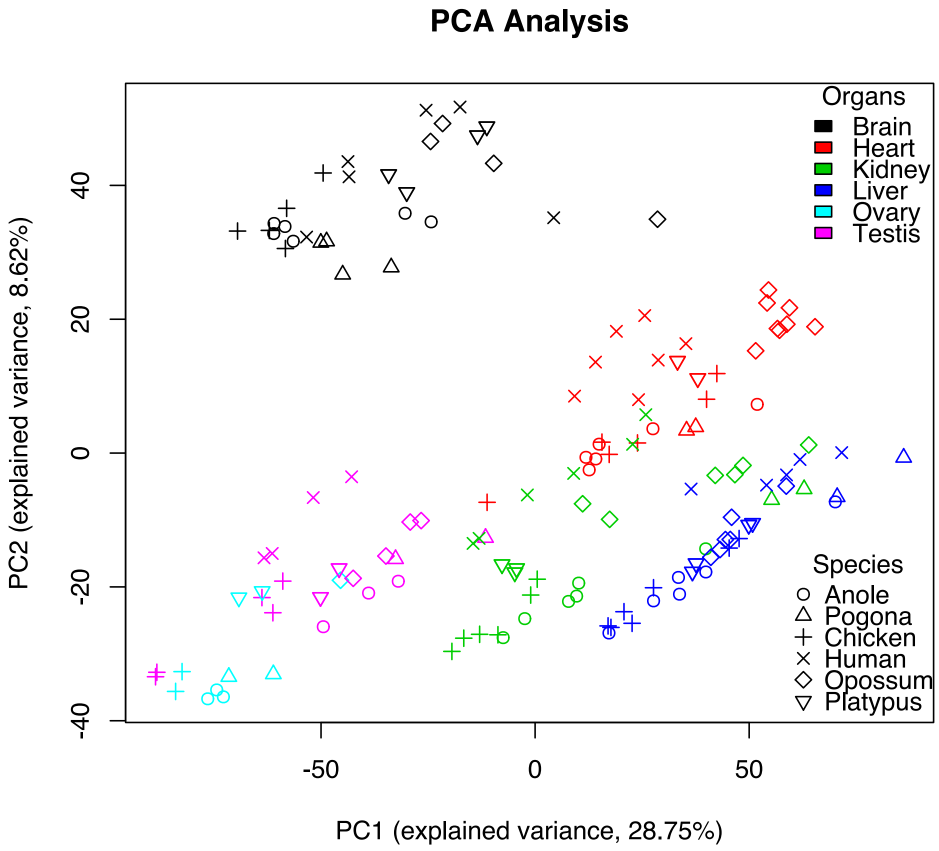

Figure S7: **Factorial map of the principal-component analysis of messenger RNA expression levels.**

This figure shows the PCA analysis of gene expression from six species (anole, bearded dragon (pogona), chicken, human, opossum and platypus) within six organs (brain, heart, kidney, liver, ovary and testis). Human samples did not include ovary. The proportion of the variance explained by the principal components is indicated in parentheses.

Supplementary Tables

Table 1: **Gene expression dataset.** Show the systematic name, common name, Gender, Tissue, layout, source, study and instrument. Gene expression that were acquired through private collaboration (not publicly available) are marked as 'Private' in the Submitter column. The Following abbreviations are used for submitters:
IH2500 = Illumina HiSeq 2500
IH2000 = Illumina HiSeq 2000
IGA IIX = Illumina Genome Analyzer IIX

Read accession(s)	Systematic Name	Common Name	Gender	Tissue	Layout	Source	Study	Instrument
SRR5412144	*Anolis carolinensis*	Anole	Female	Brain	Single	NCBI	Marin	IH2500
SRR5412145	*Anolis carolinensis*	Anole	Female	Brain	Single	NCBI	Marin	IH2500
SRR5412146	*Anolis carolinensis*	Anole	Female	Brain	Single	NCBI	Marin	IH2500
SRR5412147	*Anolis carolinensis*	Anole	Male	Brain	Single	NCBI	Marin	IH2500
SRR5412148	*Anolis carolinensis*	Anole	Male	Brain	Single	NCBI	Marin	IH2500
SRR5412149	*Anolis carolinensis*	Anole	Male	Brain	Single	NCBI	Marin	IH2500
SRR5412150	*Anolis carolinensis*	Anole	Female	Heart	Single	NCBI	Marin	IH2500
SRR5412151	*Anolis carolinensis*	Anole	Female	Heart	Single	NCBI	Marin	IH2500
SRR5412152	*Anolis carolinensis*	Anole	Female	Heart	Single	NCBI	Marin	IH2500
SRR5412153	*Anolis carolinensis*	Anole	Male	Heart	Single	NCBI	Marin	IH2500
SRR5412154	*Anolis carolinensis*	Anole	Male	Heart	Single	NCBI	Marin	IH2500
SRR5412155	*Anolis carolinensis*	Anole	Male	Heart	Single	NCBI	Marin	IH2500
SRR5412156	*Anolis carolinensis*	Anole	Female	Kidney	Single	NCBI	Marin	IH2500
SRR5412157	*Anolis carolinensis*	Anole	Female	Kidney	Single	NCBI	Marin	IH2500
SRR5412158	*Anolis carolinensis*	Anole	Female	Kidney	Single	NCBI	Marin	IH2500
SRR5412159	*Anolis carolinensis*	Anole	Male	Kidney	Single	NCBI	Marin	IH2500
SRR5412160	*Anolis carolinensis*	Anole	Male	Kidney	Single	NCBI	Marin	IH2500
SRR5412161	*Anolis carolinensis*	Anole	Male	Kidney	Single	NCBI	Marin	IH2500
SRR5412162	*Anolis carolinensis*	Anole	Female	Liver	Single	NCBI	Marin	IH2500
SRR5412163	*Anolis carolinensis*	Anole	Female	Liver	Single	NCBI	Marin	IH2500
SRR5412164	*Anolis carolinensis*	Anole	Female	Liver	Single	NCBI	Marin	IH2500
SRR5412165	*Anolis carolinensis*	Anole	Male	Liver	Single	NCBI	Marin	IH2500
SRR5412166	*Anolis carolinensis*	Anole	Male	Liver	Single	NCBI	Marin	IH2500
SRR5412167	*Anolis carolinensis*	Anole	Male	Liver	Single	NCBI	Marin	IH2500
SRR5412168	*Anolis carolinensis*	Anole	Female	Ovary	Single	NCBI	Marin	IH2500
SRR5412169	*Anolis carolinensis*	Anole	Female	Ovary	Single	NCBI	Marin	IH2500
SRR5412170	*Anolis carolinensis*	Anole	Female	Ovary	Single	NCBI	Marin	IH2500
SRR5412171	*Anolis carolinensis*	Anole	Male	Testes	Single	NCBI	Marin	IH2500
SRR5412172	*Anolis carolinensis*	Anole	Male	Testes	Single	NCBI	Marin	IH2500
SRR5412173	*Anolis carolinensis*	Anole	Male	Testes	Single	NCBI	Marin	IH2500
SRR5412242	*Gallus gallus*	Chicken	Female	Brain	Single	NCBI	Marin	IH2500
SRR5412243	*Gallus gallus*	Chicken	Male	Brain	Single	NCBI	Marin	IH2500
SRR5412244	*Gallus gallus*	Chicken	Male	Brain	Single	NCBI	Marin	IH2500
SRR306710	*Gallus gallus*	Chicken	Female	Brain	Single	NCBI	Brawand	IGA IIX
SRR306711	*Gallus gallus*	Chicken	Male	Brain	Single	NCBI	Brawand	IGA IIX
SRR5412245	*Gallus gallus*	Chicken	Female	Heart	Single	NCBI	Marin	IH2500
SRR5412246	*Gallus gallus*	Chicken	Female	Heart	Single	NCBI	Marin	IH2500
SRR5412247	*Gallus gallus*	Chicken	Male	Heart	Single	NCBI	Marin	IH2500

SRR5412248	*Gallus gallus*	Chicken	Male	Heart	Single	NCBI	Marin	IH2500
SRR306714	*Gallus gallus*	Chicken	Female	Heart	Single	NCBI	Brawand	IGA IIX
SRR306715	*Gallus gallus*	Chicken	Male	Heart	Single	NCBI	Brawand	IGA IIX
SRR5412249	*Gallus gallus*	Chicken	Female	Kidney	Single	NCBI	Marin	IH2500
SRR5412250	*Gallus gallus*	Chicken	Female	Kidney	Single	NCBI	Marin	IH2500
SRR5412251	*Gallus gallus*	Chicken	Male	Kidney	Single	NCBI	Marin	IH2500
SRR5412252	*Gallus gallus*	Chicken	Male	Kidney	Single	NCBI	Marin	IH2500
SRR306716	*Gallus gallus*	Chicken	Female	Kidney	Single	NCBI	Brawand	IGA IIX
SRR306717	*Gallus gallus*	Chicken	Male	Kidney	Single	NCBI	Brawand	IGA IIX
SRR5412253	*Gallus gallus*	Chicken	Female	Liver	Single	NCBI	Marin	IH2500
SRR5412254	*Gallus gallus*	Chicken	Female	Liver	Single	NCBI	Marin	IH2500
SRR5412255	*Gallus gallus*	Chicken	Male	Liver	Single	NCBI	Marin	IH2500
SRR5412256	*Gallus gallus*	Chicken	Male	Liver	Single	NCBI	Marin	IH2500
SRR306718	*Gallus gallus*	Chicken	Female	Liver	Single	NCBI	Brawand	IGA IIX
SRR306719	*Gallus gallus*	Chicken	Male	Liver	Single	NCBI	Brawand	IGA IIX
SRR306720	*Gallus gallus*	Chicken	Male	Liver	Single	NCBI	Brawand	IGA IIX
SRR5412257	*Gallus gallus*	Chicken	Female	Ovary	Single	NCBI	Marin	IH2500
SRR5412258	*Gallus gallus*	Chicken	Female	Ovary	Single	NCBI	Marin	IH2500
SRR5412259	*Gallus gallus*	Chicken	Male	Testis	Single	NCBI	Marin	IH2500
SRR5412260	*Gallus gallus*	Chicken	Male	Testis	Single	NCBI	Marin	IH2500
SRR306721	*Gallus gallus*	Chicken	Male	Testis	Single	NCBI	Brawand	IGA IIX
SRR306722	*Gallus gallus*	Chicken	Male	Testis	Single	NCBI	Brawand	IGA IIX
SRR306723	*Gallus gallus*	Chicken	Male	Testis	Single	NCBI	Brawand	IGA IIX
ERR753525	*Pogona Vitticeps*	Bearded dragon	Male	Brain	Paired	NCBI	Georges	IH2000
ERR413064	*Pogona Vitticeps*	Bearded dragon	Male	Brain	Paired	NCBI	Georges	IH2000
ERR753526	*Pogona Vitticeps*	Bearded dragon	Female	Brain	Paired	NCBI	Georges	IH2000
ERR413071	*Pogona Vitticeps*	Bearded dragon	Female	Brain	Paired	NCBI	Georges	IH2000
ERR413072	*Pogona Vitticeps*	Bearded dragon	Female	Heart	Paired	NCBI	Georges	IH2000
ERR413065	*Pogona Vitticeps*	Bearded dragon	Male	Heart	Paired	NCBI	Georges	IH2000
ERR413073	*Pogona Vitticeps*	Bearded dragon	Female	Kidney	Paired	NCBI	Georges	IH2000
ERR413066	*Pogona Vitticeps*	Bearded dragon	Male	Kidney	Paired	NCBI	Georges	IH2000
ERR413074	*Pogona Vitticeps*	Bearded dragon	Female	Liver	Paired	NCBI	Georges	IH2000
ERR413067	*Pogona Vitticeps*	Bearded dragon	Male	Liver	Paired	NCBI	Georges	IH2000
ERR413070	*Pogona Vitticeps*	Bearded dragon	Male	Testis	Paired	NCBI	Georges	IH2000
ERR753529	*Pogona Vitticeps*	Bearded dragon	Male	Testis	Paired	NCBI	Georges	IH2000
ERR753530	*Pogona Vitticeps*	Bearded dragon	Female	Ovary	Paired	NCBI	Georges	IH2000
ERR413082	*Pogona Vitticeps*	Bearded dragon	Female	Ovary	Paired	NCBI	Georges	IH2000
SRR5412222	*Ornithorhynchus anatinus*	Platypus	Female	Brain	Single	NCBI	Marin	IH2500
SRR5412223	*Ornithorhynchus anatinus*	Platypus	Female	Brain	Single	NCBI	Marin	IH2500
SRR5412224	*Ornithorhynchus anatinus*	Platypus	Male	Brain	Single	NCBI	Marin	IH2500
SRR5412225	*Ornithorhynchus anatinus*	Platypus	Male	Brain	Single	NCBI	Marin	IH2500
SRR306724	*Ornithorhynchus anatinus*	Platypus	Female	Brain	Single	NCBI	Brawand	IGA IIX
SRR306725	*Ornithorhynchus anatinus*	Platypus	Female	Brain	Single	NCBI	Brawand	IGA IIX
SRR306726	*Ornithorhynchus anatinus*	Platypus	Male	Brain	Single	NCBI	Brawand	IGA IIX
SRR306727	*Ornithorhynchus anatinus*	Platypus	Male	Brain	Single	NCBI	Brawand	IGA IIX
SRR5412226	*Ornithorhynchus anatinus*	Platypus	Female	Heart	Single	NCBI	Marin	IH2500

SRR5412227	*Ornithorhynchus anatinus*	Platypus	Female	Heart	Single	NCBI	Marin	IH2500
SRR5412228	*Ornithorhynchus anatinus*	Platypus	Male	Heart	Single	NCBI	Marin	IH2500
SRR5412229	*Ornithorhynchus anatinus*	Platypus	Male	Heart	Single	NCBI	Marin	IH2500
SRR306730	*Ornithorhynchus anatinus*	Platypus	Female	Heart	Single	NCBI	Brawand	IGA IIX
SRR306731	*Ornithorhynchus anatinus*	Platypus	Male	Heart	Single	NCBI	Brawand	IGA IIX
SRR5412230	*Ornithorhynchus anatinus*	Platypus	Female	Kidney	Single	NCBI	Marin	IH2500
SRR5412231	*Ornithorhynchus anatinus*	Platypus	Female	Kidney	Single	NCBI	Marin	IH2500
SRR5412232	*Ornithorhynchus anatinus*	Platypus	Male	Kidney	Single	NCBI	Marin	IH2500
SRR5412233	*Ornithorhynchus anatinus*	Platypus	Male	Kidney	Single	NCBI	Marin	IH2500
SRR306732	*Ornithorhynchus anatinus*	Platypus	Female	Kidney	Single	NCBI	Brawand	IGA IIX
SRR306733	*Ornithorhynchus anatinus*	Platypus	Male	Kidney	Single	NCBI	Brawand	IGA IIX
SRR306734	*Ornithorhynchus anatinus*	Platypus	Male	Kidney	Single	NCBI	Brawand	IGA IIX
SRR5412234	*Ornithorhynchus anatinus*	Platypus	Female	Liver	Single	NCBI	Marin	IH2500
SRR5412235	*Ornithorhynchus anatinus*	Platypus	Female	Liver	Single	NCBI	Marin	IH2500
SRR5412236	*Ornithorhynchus anatinus*	Platypus	Male	Liver	Single	NCBI	Marin	IH2500
SRR5412237	*Ornithorhynchus anatinus*	Platypus	Male	Liver	Single	NCBI	Marin	IH2500
SRR306735	*Ornithorhynchus anatinus*	Platypus	Female	Liver	Single	NCBI	Brawand	IGA IIX
SRR306736	*Ornithorhynchus anatinus*	Platypus	Female	Liver	Single	NCBI	Brawand	IGA IIX
SRR306737	*Ornithorhynchus anatinus*	Platypus	Male	Liver	Single	NCBI	Brawand	IGA IIX
SRR306738	*Ornithorhynchus anatinus*	Platypus	Male	Liver	Single	NCBI	Brawand	IGA IIX
SRR5412238	*Ornithorhynchus anatinus*	Platypus	Female	Ovary	Single	NCBI	Marin	IH2500
SRR5412239	*Ornithorhynchus anatinus*	Platypus	Female	Ovary	Single	NCBI	Marin	IH2500
SRR5412240	*Ornithorhynchus anatinus*	Platypus	Male	Testis	Single	NCBI	Marin	IH2500
SRR5412241	*Ornithorhynchus anatinus*	Platypus	Male	Testis	Single	NCBI	Marin	IH2500
SRR306739	*Ornithorhynchus anatinus*	Platypus	Male	Testis	Single	NCBI	Brawand	IGA IIX
SRR306741	*Ornithorhynchus anatinus*	Platypus	Male	Testis	Single	NCBI	Brawand	IGA IIX
SRR5412205	*Monodelphis domestica*	Opossum	Female	Brain	Single	NCBI	Marin	IH2500
SRR5412206	*Monodelphis domestica*	Opossum	Male	Brain	Single	NCBI	Marin	IH2500
SRR306742	*Monodelphis domestica*	Opossum	Female	Brain	Single	NCBI	Brawand	IGA IIX
SRR306743	*Monodelphis domestica*	Opossum	Female	Brain	Single	NCBI	Brawand	IGA IIX
SRR306744	*Monodelphis domestica*	Opossum	Male	Brain	Single	NCBI	Brawand	IGA IIX
SRR5412207	*Monodelphis domestica*	Opossum	Female	Heart	Single	NCBI	Marin	IH2500
SRR5412208	*Monodelphis domestica*	Opossum	Female	Heart	Single	NCBI	Marin	IH2500
SRR5412209	*Monodelphis domestica*	Opossum	Male	Heart	Single	NCBI	Marin	IH2500
SRR5412210	*Monodelphis domestica*	Opossum	Male	Heart	Single	NCBI	Marin	IH2500
SRR306747	*Monodelphis domestica*	Opossum	Female	Heart	Single	NCBI	Brawand	IGA IIX
SRR306748	*Monodelphis domestica*	Opossum	Female	Heart	Single	NCBI	Brawand	IGA IIX
SRR306749	*Monodelphis domestica*	Opossum	Male	Heart	Single	NCBI	Brawand	IGA IIX
SRR306750	*Monodelphis domestica*	Opossum	Male	Heart	Single	NCBI	Brawand	IGA IIX
SRR5412211	*Monodelphis domestica*	Opossum	Female	Kidney	Single	NCBI	Marin	IH2500
SRR5412212	*Monodelphis domestica*	Opossum	Female	Kidney	Single	NCBI	Marin	IH2500
SRR5412213	*Monodelphis domestica*	Opossum	Male	Kidney	Single	NCBI	Marin	IH2500
SRR5412214	*Monodelphis domestica*	Opossum	Male	Kidney	Single	NCBI	Marin	IH2500
SRR306751	*Monodelphis domestica*	Opossum	Female	Kidney	Single	NCBI	Brawand	IGA IIX
SRR306752	*Monodelphis domestica*	Opossum	Male	Kidney	Single	NCBI	Brawand	IGA IIX
SRR5412215	*Monodelphis domestica*	Opossum	Female	Liver	Single	NCBI	Marin	IH2500
SRR5412216	*Monodelphis domestica*	Opossum	Female	Liver	Single	NCBI	Marin	IH2500
SRR5412217	*Monodelphis domestica*	Opossum	Male	Liver	Single	NCBI	Marin	IH2500
SRR5412218	*Monodelphis domestica*	Opossum	Male	Liver	Single	NCBI	Marin	IH2500
SRR306753	*Monodelphis domestica*	Opossum	Female	Liver	Single	NCBI	Brawand	IGA IIX

SRR306754	*Monodelphis domestica*	Opossum	Male	Liver	Single	NCBI	Brawand	IGA IIX
SRR5412219	*Monodelphis domestica*	Opossum	Female	Ovary	Single	NCBI	Marin	IH2500
SRR5412220	*Monodelphis domestica*	Opossum	Male	Testis	Single	NCBI	Marin	IH2500
SRR5412221	*Monodelphis domestica*	Opossum	Male	Testis	Single	NCBI	Marin	IH2500
SRR306755	*Monodelphis domestica*	Opossum	Male	Testis	Single	NCBI	Brawand	IGA IIX
SRR306756	*Monodelphis domestica*	Opossum	Male	Testis	Single	NCBI	Brawand	IGA IIX
SRR5412174	*Homo sapiens*	Human	Female	Brain	Single	NCBI	Marin	IH2500
SRR5412175	*Homo sapiens*	Human	Male	Brain	Single	NCBI	Marin	IH2500
SRR306838	*Homo sapiens*	Human	Female	Brain	Single	NCBI	Brawand	IGA IIX
SRR306839	*Homo sapiens*	Human	Male	Brain	Single	NCBI	Brawand	IGA IIX
SRR306841	*Homo sapiens*	Human	Male	Brain	Single	NCBI	Brawand	IGA IIX
SRR306843	*Homo sapiens*	Human	Male	Brain	Single	NCBI	Brawand	IGA IIX
SRR5412176	*Homo sapiens*	Human	Female	Heart	Paired	NCBI	Marin	IH2500
SRR5412177	*Homo sapiens*	Human	Male	Heart	Single	NCBI	Marin	IH2500
SRR5412178	*Homo sapiens*	Human	Male	Heart	Paired	NCBI	Marin	IH2500
SRR306847	*Homo sapiens*	Human	Female	Heart	Single	NCBI	Brawand	IGA IIX
SRR306848	*Homo sapiens*	Human	Male	Heart	Single	NCBI	Brawand	IGA IIX
SRR306849	*Homo sapiens*	Human	Male	Heart	Single	NCBI	Brawand	IGA IIX
SRR306850	*Homo sapiens*	Human	Male	Heart	Single	NCBI	Brawand	IGA IIX
SRR5412179	*Homo sapiens*	Human	Female	Kidney	Single	NCBI	Marin	IH2500
SRR5412180	*Homo sapiens*	Human	Male	Kidney	Single	NCBI	Marin	IH2500
SRR5412181	*Homo sapiens*	Human	Male	Kidney	Single	NCBI	Marin	IH2500
SRR306851	*Homo sapiens*	Human	Female	Kidney	Single	NCBI	Brawand	IGA IIX
SRR306852	*Homo sapiens*	Human	Male	Kidney	Single	NCBI	Brawand	IGA IIX
SRR306853	*Homo sapiens*	Human	Male	Kidney	Single	NCBI	Brawand	IGA IIX
SRR5412182	*Homo sapiens*	Human	Female	Liver	Single	NCBI	Marin	IH2500
SRR5412183	*Homo sapiens*	Human	Male	Liver	Single	NCBI	Marin	IH2500
SRR306854	*Homo sapiens*	Human	Male	Liver	Single	NCBI	Brawand	IGA IIX
SRR306855	*Homo sapiens*	Human	Male	Liver	Single	NCBI	Brawand	IGA IIX
SRR306856	*Homo sapiens*	Human	Male	Liver	Single	NCBI	Brawand	IGA IIX
SRR5412184	*Homo sapiens*	Human	Male	Testis	Single	NCBI	Marin	IH2500
SRR5412185	*Homo sapiens*	Human	Male	Testis	Single	NCBI	Marin	IH2500
SRR306857	*Homo sapiens*	Human	Male	Testes	Single	NCBI	Brawand	IGA IIX
SRR306858	*Homo sapiens*	Human	Male	Testes	Single	NCBI	Brawand	IGA IIX

Table 2: **Assembly dataset**. Shows the systematic name, common name, genome version, source and submitter for all the genomes tested with our *ab initio* method.
The Following abbreviations are used for submitters:
Genome Sequencing Platform, The Genome Assembly Team = GAT;
Genome Reference Consortium = GRC;
International Chicken Genome Consortium = ICGS;
Washington University = WashU.

No	Systematic Name	Common Name	RefSeq Assembly Accession	Source	Submitter
1	*Homo sapiens*	Human	GCF_000001405.25	NCBI	GRC
2	*Pogona Vitticeps*	Bearded Dragon	GCF_900067755.1	NCBI	BRAEMBL
3	*Anolis Carollinensis*	Anole lizard	GCF_000090745.1	NCBI	Broad
4	*Gallus gallus*	Chicken	GCF_000002315.3	NCBI	ICGS
5	*Monodelphis domestica*	Opossum	GCF_000002295.2	NCBI	GAT
6	*Ornithorhynchus anatinus*	Platypus	GCF_000002275.2	NCBI	WashU

Table 3: **Comparison of orthologs with ssTE *vs* orthologs with nsTE and ∅ TE**. Shows the number of sample genes used in the bootstrap approach. Test sample is ortholog genes containing recent species-specific TE (ssTE), reference sample is ortholog genes with no ssTE.

	**Chicken**		**Anole**		**Bearded dragon**		**Platypus**		**Opossum**		**Human**	
	Test	Reference	Test	Reference	Test	Reference	Test	Reference	Test	Reference	Test	Reference
LINE	1,580	5,015	4,135	2,640	3,613	2,982	1,854	4,741	3,274	3,321	2,048	4,547
SINE	0	NA	1,566	5,029	5,660	935	513	NA	317	NA	3,388	3,207
ERV	143	NA	2,340	4,255	104	NA	16	NA	3,064	3,531	994	5,601
DNA	5	NA	3,436	3,159	496	NA	6	NA	236	NA	45	NA

Table 4: **Comparison of orthologs with nsTE *vs* orthologs with ssTE and ∅ TE**. Shows the number of sample genes used in the bootstrap approach. Test sample is ortholog genes containing non-species specific TE (nsTE), reference sample is ortholog genes with no nsTE.

	**Chicken**		**Anole**		**Bearded dragon**		**Platypus**		**Opossum**		**Human**	
	Test	Reference	Test	Reference	Test	Reference	Test	Reference	Test	Reference	Test	Reference
LINE	4,320	2,275	2,221	4,374	2,805	3,790	4,516	2,079	3,013	3,582	4,340	2,255
SINE	1,174	5,421	4,369	2,226	4,667	1,928	5,830	765	6,076	NA	3,070	3,525
ERV	5,797	NA	3,652	2,943	6,106	NA	5,374	NA	2,871	3,724	5,470	1,125
DNA	5,894	NA	3,066	3,529	5,931	NA	5,525	NA	5,819	NA	6,455	NA

Table 5: **Comparison of non-orthologs with ssTE *vs* non-orthologs with nsTE and ∅ TE**. Shows the number of sample genes used in bootstrap approach. Test sample is non-ortholog genes containing recent species-specific TE (ssTE), reference sample is non-ortholog genes with no ssTE.

	**Chicken**		**Anole**		**Bearded dragon**		**Platypus**		**Opossum**		**Human**	
	Test	Reference	Test	Reference	Test	Reference	Test	Reference	Test	Reference	Test	Reference
LINE	1,488	9,025	8,337	10,988	5,671	9,728	2,677	16,844	5,025	12,279	6,065	45,076
SINE	0	NA	2,670	16,655	1,203	14,196	553	NA	413	NA	8,603	42,538
ERV	211	NA	4,528	14,797	142	NA	24	NA	4,439	12,865	3,401	47,740
DNA	11	NA	5,593	13,372	560	NA	5	NA	344	NA	220	NA

Table 6: **Comparison of non-orthologs with nsTE *vs* non-orthologs with ssTE and ∅ TE**. Shows the number of sample genes used in the bootstrap approach. Test sample is non-ortholog genes containing non-species specific TE (nsTE), reference sample is non-ortholog genes with no nsTE.

	**Chicken**		**Anole**		**Bearded dragon**		**Platypus**		**Opossum**		**Human**	
	Test	Reference	Test	Reference	Test	Reference	Test	Reference	Test	Reference	Test	Reference
LINE	6,402	4,111	8,186	11,139	8,428	6,971	14,113	5,408	9,423	7,881	32,875	18,266
SINE	1,191	9,322	11,472	7,853	9,147	6,252	16,175	3,346	13,241	4,063	32,869	18,272
ERV	7,453	3,060	8,690	10,635	12,671	2,728	9,778	9,743	7,360	9,944	34,177	18,202
DNA	7,397	3,116	11,320	8,005	13,190	2,209	9,878	NA	10,232	7,072	32,939	16,964

Table 7: **Summary of orthologs/non-orthologs with nsTE, orthologs/non-orthologs with ssTE and orthologs/non-orthologs with ∅ TE.** Shows the number of genes with ssTE, nsTE and ∅ TE.
ortho: Ortholog
non-ortho: Non-ortholog

	**Chicken**		**Anole**		**Bearded dragon**		**Platypus**		**Opossum**		**Human**	
	ortho	non-ortho	ortho	non-ortho	ortho	non-ortho	ortho	non-ortho	ortho	non-ortho	ortho	non-ortho
ssTE	1,638	1,589	5,145	11,458	3,825	6,109	2,057	3,235	4,077	6,787	4,115	13,316
nsTE	6,552	10,017	6,584	19,255	6,585	15,273	6,570	19,197	6,565	17,099	6,594	50,878
∅ TE	43	497	11	70	10	127	25	325	30	206	1	264

Table 8: **Difference in the gene expression of orthologs/non-orthologs with a TE insertion.** Shows the species, TE element, Tissue, gene expression comparison sets , lowest gene expression level, median gene expression level, highest gene expression level, 95% CI lowest gene expression, 95% CI highest expression level and the significance indicator. TPM counts were log2 transformed.

Species	Element	Tissue	Data	lwr	med	upr	lwr95	upr95	Sig
Platypus	LINE	Heart	ssTE-ortholog	-0.859	-0.443	-0.045	-0.769	-0.131	T
Bearded dragon	LINE	Heart	ssTE-ortholog	-0.704	-0.387	-0.064	-0.626	-0.147	T
Bearded dragon	LINE	Kidney	ssTE-ortholog	-0.815	-0.485	-0.160	-0.732	-0.242	T
Bearded dragon	LINE	Liver	ssTE-ortholog	-0.864	-0.525	-0.188	-0.771	-0.276	T
Bearded dragon	LINE	Ovary	ssTE-ortholog	-0.975	-0.629	-0.278	-0.883	-0.373	T
Bearded dragon	LINE	Testes	ssTE-ortholog	-0.677	-0.369	-0.041	-0.599	-0.129	T
Bearded dragon	SINE	Heart	ssTE-ortholog	-0.946	-0.584	-0.218	-0.851	-0.322	T
Bearded dragon	SINE	Kidney	ssTE-ortholog	-1.035	-0.672	-0.325	-0.933	-0.406	T
Bearded dragon	SINE	Liver	ssTE-ortholog	-0.965	-0.566	-0.194	-0.851	-0.283	T
Bearded dragon	SINE	Ovary	ssTE-ortholog	-1.022	-0.592	-0.212	-0.901	-0.300	T
Bearded dragon	SINE	Testes	ssTE-ortholog	-0.939	-0.601	-0.259	-0.854	-0.351	T
Chicken	LINE	Brain	ssTE-ortholog	0.013	0.323	0.667	0.089	0.579	T
Anole	LINE	Heart	ssTE-ortholog	-0.792	-0.444	-0.112	-0.701	-0.197	T
Anole	LINE	Kidney	ssTE-ortholog	-0.830	-0.459	-0.128	-0.724	-0.212	T
Anole	LINE	Liver	ssTE-ortholog	-0.832	-0.481	-0.120	-0.743	-0.211	T
Anole	LINE	Ovary	ssTE-ortholog	-0.730	-0.402	-0.087	-0.647	-0.168	T
Anole	SINE	Heart	ssTE-ortholog	-0.847	-0.509	-0.191	-0.758	-0.266	T
Anole	SINE	Kidney	ssTE-ortholog	-0.824	-0.506	-0.185	-0.745	-0.267	T
Anole	SINE	Ovary	ssTE-ortholog	-0.838	-0.493	-0.170	-0.751	-0.252	T
Anole	SINE	Testes	ssTE-ortholog	-0.762	-0.459	-0.154	-0.684	-0.232	T
Anole	ERV	Heart	ssTE-ortholog	-0.760	-0.426	-0.104	-0.669	-0.187	T
Anole	ERV	Kidney	ssTE-ortholog	-0.968	-0.653	-0.348	-0.886	-0.425	T
Anole	ERV	Liver	ssTE-ortholog	-1.011	-0.680	-0.347	-0.924	-0.433	T
Anole	ERV	Ovary	ssTE-ortholog	-0.709	-0.391	-0.066	-0.628	-0.151	T
Human	LINE	Kidney	ssTE-ortholog	-1.179	-0.746	-0.041	-1.079	-0.126	T
Human	LINE	Liver	ssTE-ortholog	-1.283	-0.738	-0.116	-1.179	-0.216	T
Human	LINE	Testes	ssTE-ortholog	-0.917	-0.562	-0.221	-0.840	-0.296	T
Human	SINE	Brain	ssTE-ortholog	0.119	0.429	0.750	0.196	0.671	T
Human	SINE	Testes	ssTE-ortholog	0.274	0.619	0.933	0.357	0.858	T
Bearded dragon	LINE	Kidney	ssTE-nonOrtholog	-0.836	-0.433	-0.063	-0.695	-0.172	T
Bearded dragon	LINE	Ovary	ssTE-nonOrtholog	-0.929	-0.510	-0.091	-0.797	-0.226	T
Bearded dragon	SINE	Heart	ssTE-nonOrtholog	-0.859	-0.498	-0.136	-0.748	-0.250	T
Bearded dragon	SINE	Kidney	ssTE-nonOrtholog	-0.846	-0.463	-0.074	-0.725	-0.199	T
Bearded dragon	SINE	Ovary	ssTE-nonOrtholog	-1.063	-0.616	-0.200	-0.933	-0.334	T
Bearded dragon	SINE	Testes	ssTE-nonOrtholog	-0.720	-0.375	-0.026	-0.619	-0.136	T
Chicken	LINE	Brain	ssTE-nonOrtholog	0.008	0.388	0.832	0.125	0.694	T
Anole	ERV	Heart	ssTE-nonOrtholog	-0.855	-0.456	-0.076	-0.730	-0.191	T
Anole	ERV	Kidney	ssTE-nonOrtholog	-0.764	-0.378	-0.020	-0.640	-0.127	T
Anole	ERV	Liver	ssTE-nonOrtholog	-0.865	-0.458	-0.062	-0.737	-0.191	T
Anole	ERV	Ovary	ssTE-nonOrtholog	-0.876	-0.461	-0.073	-0.743	-0.194	T
Human	SINE	Brain	ssTE-nonOrtholog	0.350	1.099	1.629	0.470	1.478	T
Human	SINE	Heart	ssTE-nonOrtholog	0.206	1.005	1.538	0.315	1.375	T
Human	SINE	Kidney	ssTE-nonOrtholog	0.386	1.109	1.590	0.511	1.451	T
Human	SINE	Liver	ssTE-nonOrtholog	0.085	0.759	1.292	0.218	1.147	T
Human	SINE	Testes	ssTE-nonOrtholog	0.685	1.310	1.765	0.815	1.621	T
Platypus	SINE	Liver	nsTE-ortholog	0.089	0.622	1.168	0.251	1.001	T
Platypus	SINE	Ovary	nsTE-ortholog	0.027	0.473	0.924	0.162	0.780	T
Platypus	SINE	Testes	nsTE-ortholog	0.114	0.567	1.031	0.250	0.890	T
Bearded dragon	LINE	Heart	nsTE-ortholog	0.009	0.391	0.786	0.134	0.649	T

Bearded dragon	LINE	Kidney	nsTE-ortholog	0.050	0.428	0.795	0.172	0.687	T
Bearded dragon	LINE	Liver	nsTE-ortholog	0.125	0.523	0.916	0.250	0.790	T
Bearded dragon	LINE	Ovary	nsTE-ortholog	0.186	0.577	0.972	0.300	0.840	T
Bearded dragon	LINE	Testes	nsTE-ortholog	0.007	0.368	0.733	0.125	0.610	T
Chicken	SINE	Heart	nsTE-ortholog	-1.039	-0.624	-0.250	-0.905	-0.365	T
Chicken	SINE	Kidney	nsTE-ortholog	-1.134	-0.743	-0.356	-1.012	-0.472	T
Chicken	SINE	Liver	nsTE-ortholog	-1.424	-0.935	-0.451	-1.272	-0.583	T
Chicken	SINE	Ovary	nsTE-ortholog	-1.051	-0.685	-0.340	-0.935	-0.447	T
Chicken	SINE	Testes	nsTE-ortholog	-1.025	-0.715	-0.412	-0.925	-0.508	T
Anole	LINE	Heart	nsTE-ortholog	0.149	0.514	0.895	0.260	0.777	T
Anole	LINE	Kidney	nsTE-ortholog	0.212	0.567	0.974	0.320	0.844	T
Anole	LINE	Liver	nsTE-ortholog	0.175	0.578	0.978	0.303	0.852	T
Anole	LINE	Ovary	nsTE-ortholog	0.170	0.534	0.919	0.282	0.795	T
Anole	SINE	Heart	nsTE-ortholog	0.035	0.393	0.758	0.148	0.643	T
Anole	SINE	Ovary	nsTE-ortholog	0.109	0.474	0.836	0.222	0.726	T
Anole	SINE	Testes	nsTE-ortholog	0.084	0.431	0.773	0.196	0.665	T
Human	LINE	Liver	nsTE-ortholog	0.033	0.584	1.159	0.155	1.002	T
Human	LINE	Testes	nsTE-ortholog	0.102	0.474	0.844	0.209	0.732	T
Human	SINE	Testes	nsTE-ortholog	-0.776	-0.411	-0.063	-0.664	-0.169	T
Bearded dragon	LINE	Ovary	nsTE-nonOrtholog	0.012	0.446	0.879	0.155	0.743	T
Bearded dragon	DNA	Kidney	nsTE-nonOrtholog	0.023	0.464	0.892	0.186	0.751	T
Chicken	SINE	Kidney	nsTE-nonOrtholog	-0.915	-0.502	-0.102	-0.775	-0.234	T
Chicken	SINE	Liver	nsTE-nonOrtholog	-1.459	-0.960	-0.421	-1.292	-0.585	T
Chicken	SINE	Ovary	nsTE-nonOrtholog	-0.913	-0.522	-0.086	-0.781	-0.255	T
Opossum	SINE	Ovary	nsTE-nonOrtholog	0.053	0.449	0.830	0.178	0.709	T
Human	SINE	Brain	nsTE-nonOrtholog	-0.955	-0.481	-0.071	-0.803	-0.198	T
Human	SINE	Heart	nsTE-nonOrtholog	-0.937	-0.451	-0.033	-0.772	-0.156	T
Human	SINE	Kidney	nsTE-nonOrtholog	-0.955	-0.485	-0.054	-0.804	-0.191	T
Human	SINE	Testes	nsTE-nonOrtholog	-0.879	-0.473	-0.044	-0.740	-0.202	T
Human	ERV	Brain	nsTE-nonOrtholog	0.382	1.149	1.651	0.521	1.482	T
Human	ERV	Heart	nsTE-nonOrtholog	0.278	1.097	1.638	0.437	1.469	T
Human	ERV	Kidney	nsTE-nonOrtholog	0.631	1.235	1.806	0.787	1.611	T
Human	ERV	Liver	nsTE-nonOrtholog	0.493	1.159	1.683	0.636	1.521	T
Human	ERV	Testes	nsTE-nonOrtholog	0.624	1.321	1.815	0.765	1.656	T
Human	DNA	Testes	nsTE-nonOrtholog	0.020	0.433	0.891	0.162	0.717	T
